# The INSPIRE Bio-Resource Research Platform for Healthy Aging and Geroscience: Focus on the Human Translational Research Cohort (The INSPIRE-T Cohort)

**DOI:** 10.14283/jfa.2020.38

**Published:** 2020-07-10

**Authors:** Sophie Guyonnet, Y. Rolland, C. Takeda, P.-J. Ousset, I. Ader, N. Davezac, C. Dray, N. Fazilleau, P. Gourdy, R. Liblau, A. Parini, P. Payoux, L. Pénicaud, C. Rampon, P. Valet, N. Vergnolle, S. Andrieu, P. De Souto Barreto, L. Casteilla, B. Vellas, INSPIRE Platform Group

**Affiliations:** 1 Inserm UMR 1027, Toulouse, France; 2 University of Toulouse III, Toulouse, France; 3 Gérontopôle, Department of Geriatrics, CHU Toulouse, Toulouse, France; 4 STROMALab, Etablissement Français du Sang-Occitanie (EFS), Inserm 1031, University of Toulouse, National Veterinary School of Toulouse (ENVT), ERL5311 CNRS, Toulouse, France; 5 Centre de Recherches sur la Cognition Animale (CRCA), Centre de Biologie Intégrative (CBI), Université de Toulouse, CNRS, UPS, Toulouse, France; 6 Institut des Maladies Métaboliques et Cardiovasculaires, Inserm/Université Paul Sabatier UMR 1048, I2MC 1 avenue Jean Poulhès, BP 84225, 31432 Toulouse Cedex 4, France; 7 Centre de Physiopathologie Toulouse Purpan, INSERM/CNRS/UPS UMR 1043, University of Toulouse III, Toulouse, France; 8 ToNIC, Toulouse NeuroImaging Center, Université de Toulouse, Inserm, UPS, Toulouse, France; 9 IRSD, Université de Toulouse, INSERM, INRA, ENVT, UPS, U1220, CHU Purpan, CS60039, 31024 Toulouse, France; 10 Department of Epidemiology and Public Health, CHU Toulouse, Toulouse, France; 11 Gérontopôle, Institute of Aging,, CHU Toulouse, Toulouse, France

**Keywords:** Gerosciences, integrated care, biological aging, intrinsic capacity, biology of aging, translational research on aging

## Abstract

**Background:**

The Geroscience field focuses on the core biological mechanisms of aging, which are involved in the onset of age-related diseases, as well as declines in intrinsic capacity (IC) (body functions) leading to dependency. A better understanding on how to measure the true age of an individual or biological aging is an essential step that may lead to the definition of putative markers capable of predicting healthy aging.

**Objectives:**

The main objective of the INStitute for Prevention healthy aging and medicine Rejuvenative (INSPIRE) Platform initiative is to build a program for Geroscience and healthy aging research going from animal models to humans and the health care system. The specific aim of the INSPIRE human translational cohort (INSPIRE-T cohort) is to gather clinical, digital and imaging data, and perform relevant and extensive biobanking to allow basic and translational research on humans.

**Methods:**

The INSPIRE-T cohort consists in a population study comprising 1000 individuals in Toulouse and surrounding areas (France) of different ages (20 years or over — no upper limit for age) and functional capacity levels (from robustness to frailty, and even dependency) with follow-up over 10 years. Diversified data are collected annually in research facilities or at home according to standardized procedures. Between two annual visits, IC domains are monitored every 4-month by using the ICOPE Monitor app developed in collaboration with WHO. Once IC decline is confirmed, participants will have a clinical assessment and blood sampling to investigate markers of aging at the time IC declines are detected. Biospecimens include blood, urine, saliva, and dental plaque that are collected from all subjects at baseline and then, annually. Nasopharyngeal swabs and cutaneous surface samples are collected in a large subgroup of subjects every two years. Feces, hair bulb and skin biopsy are collected optionally at the baseline visit and will be performed again during the longitudinal follow up.

**Expected Results:**

Recruitment started on October 2019 and is expected to last for two years. Bio-resources collected and explored in the INSPIRE-T cohort will be available for academic and industry partners aiming to identify robust (set of) markers of aging, age-related diseases and IC evolution that could be pharmacologically or non-pharmacologically targetable. The INSPIRE-T will also aim to develop an integrative approach to explore the use of innovative technologies and a new, function and person-centered health care pathway that will promote a healthy aging.

**Electronic Supplementary Material:**

Supplementary material is available for this article at 10.14283/jfa.2020.38 and is accessible for authorized users.

## Introduction

Aging is an important risk factor for several adverse health outcomes, particularly chronic and metabolic diseases and intrinsic capacity (IC) decline. Since chronological age differs from biological aging, operationally defining biological aging is an essential aspect to understand the interplay between aging and health outcomes. Individuals progress differently in the aging process (“normal” aging vs “accelerated” aging), which means, biological aging is a heterogeneous process. In this context, we need to develop researches to identify biomarkers of aging and healthy aging, and know how to measure biological aging. According to the Geroscience field, understanding aging and the links with age-related diseases would contribute to prevent and/or delay the onset of various diseases and the decline in IC domains, in particular in the six operational IC domains crucial for independent living defined by the World Health Organization (WHO) (mobility, cognition, psychological, vitality, hearing and vision capacities) (1–3).

The WHO has recently published the Integrated Care for Older People, ICOPE handbook Guidance, to support Healthy Aging and to propose to health-care providers appropriate approaches to detect and manage declines in IC. With this integrated and individualized approach, WHO aims to reduce the number of dependent people by 15 million Worldwide by 2025 (4–7).

Studying concomitantly biomarkers of aging and the natural history of IC evolution in people of different ages and functional status is to date very challenging to understand the relation between biological aging and health outcomes. In this context, the INSPIRE program was built to foster research in the field of Geroscience and healthy aging. INSPIRE is a research program dedicated to biological and healthy aging, aimed at constituting a bio-resource platform going from animals to humans, from cells to individuals, from research to clinical care. INSPIRE will provide clinical, biological and technological resources for research and development on aging. The resources will be open to both academic and industry worlds in order to promote healthy aging and prevent dependency. It is a public-private initiative that brings together internationally recognized experts from basic and translational science (in particular, in the fields of immunology, metabolism, neurosciences and mesenchymal stem/stroma cells), clinical gerontology (i.e., researchers, but also physicians and nurses involved in clinical care), primary care and public health (8).

One of the main challenges of INSPIRE is to identify markers capable of determining biological aging with the implementation of human and animal cohorts. The INSPIRE Human Translational Cohort (INSPIRE-T cohort) will recruit about 1000 individuals of several chronological ages (from 20 years to 100+) and functional capacity levels (from robust to frail, and even disabled) with baseline and follow-up biological, clinical, imaging and digital data over 10 years. These data should allow us to explore and identify a set of biomarkers of aging, age-related diseases and IC evolution. In addition, the INSPIRE-T cohort aims: i/ to test the feasibility and acceptability of a new app for smartphone and tablet developed to monitor the six IC domains (locomotion, cognition, vision, hearing, vitality/nutrition, psychological status) according to the WHO recommendations; and ii/ to explore the development of digital markers of aging. This paper describes the study design of the INSPIRE-T cohort.

## Material and Methods

### Study design

The INSPIRE-T cohort, started in October 2019, is a 10-year observational study. The study population will consist of 1000 subjects recruited in the city of Toulouse and surrounding areas, South-Western France, and covering the age range of 20 years and over (no upper limit for age). Several follow-up visits will be regularly scheduled during the 10-year period of this study. Additional visits will be conditioned by the remote monitoring of IC and the onset of other major clinical conditions.

At baseline, and then once a year, diversified data (clinical, digital, imaging) and biospecimens (blood, urine, saliva and dental plaques) are collected following standardized procedures. Data collection is performed in the Clinical Research Center (CRC) of the Gerontopole — CHU Toulouse. It can also be performed in participants’ home (for more frail and disabled volunteers), or in selected Gerontopole’s collaborating centers by a mobile research team trained by the CRC. Participants are assessed by appropriately trained clinical research members.

Between yearly waves of data collection, participants are asked to record major clinical information, including adverse events (e.g. new diagnosis, SARS COV-2 diagnosis, influenza, fracture …), medical consultations, hospitalizations, and changes in the drug prescription every 4-months. They also have the six IC domains monitored (with or without the help of a caregiver) (ICOPE program Step 1) (4,7) in either the application developed in collaboration with WHO (ICOPE Monitor app) or a web platform; or through a phone call by a Gerontopole’s trained research nurse. When declines are detected in the ICOPE Step 1, a phone call is organized by the research nurse within one week to confirm this decline and to investigate the causes in collaboration with the medical research team. Once an IC decline is confirmed, participants have a thorough clinical assessment following the recommendations of the ICOPE Step 2 (4,7) and blood sampling (data are collected by research nurses in a home visit or at the research facility). Such information will enable us to investigate the response of some markers of aging at the time declines are detected. The clinical assessments and biomarkers’ exploration also allow us to propose a personalized prevention care plan to maintain function according to the recommendations from the WHO ICOPE program for usual care (ICOPE Step 3) (4,7).

**Figure 1:**
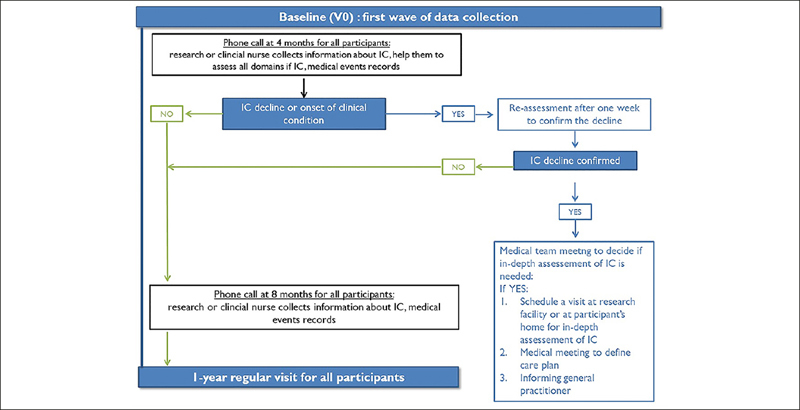
INSPIRE-T study procedures over one year. The remote monitoring of intrinsic capacity will last the whole length of this research study, i.e., up to ten years

Participants are trained to monitor their IC during the baseline visit by the Gerontopole’ research team. The remote monitoring of IC will last the whole length of this research study, i.e., up to ten years. The figure 1 shows schematically the study procedures over one year. Table 1 describes the study flow chart with all data collected at each time point during the follow-up.

**Table 1:** The INSPIRE Human Translational Research cohort flow-chart

	**Inclusion**	**Every 4-month**	**Yearly Visits**	**Every 2 years**
Information note	✓			
Informed consent	✓			
Eligibility criteria checked	✓			
Demographic information	✓			
*Physical examinations*:				
Medical history	✓		✓	
Medication	✓	✓	✓	
Vaccination	✓	✓	✓	
Current diseases	✓	✓	✓	
BMI, waist & hip circumferences	✓		✓	
Heart rate, blood pressure	✓		✓	
Self-reported visceral pain	✓		✓	
Skin elasticity (cutometer measurement)	✓		✓	
Cutaneous itching/pruritus	✓		✓	
Lifestyle information (physical activity, sedentarity time, smoking, alcohol consumption, solar exposure)	✓		✓	
Frailty Fried phenotype	✓		✓	
Functional status (ADL, IADL)	✓		✓	
Cognitive status (MMSE, cognitive composite score^a^)	✓		✓	
Physical performance (SPPB, chair rise test (30 seconds))	✓		✓	
Depressive symptoms (PHQ-9)	✓		✓	
Nutritional status (MNA, food frequency questionnaire)	✓		✓	
Oral status (OHAT)	✓		✓	
Participant-reported outcomes for cognition (CFI) and mobility, fatigue and social isolation (PROMIS)	✓		✓	
Objective physical activity and sleep parameters (activPAL accelerometer)	✓		✓	
Vision assessment (WHO simple eye chart, Amsler grid)	✓		✓	
ICOPE Monitor app (IC domains)	✓	✓	✓	
Adverse events (e.g. new diagnosis, SARS COV-2 diagnosis, influenza, fracture, …)		✓		
*Other examinations*:				
DXA	✓		✓	
MRP	✓			✓
VO^2^max with blood sampling / maximal aerobic power^b^	✓			✓
Isokinetic muscle strength^b^	✓			✓
Patient-reported outcome for sarcopenia (SAR-Qol)^b^	✓			✓

a. The cognitive composite score will be realized only in people lower than 70 years; b. Other examinations are proposed to a limited number of participants in a volunteer basis

To ensure quality of data collected, standard operating procedures are implemented covering subject’s recruitment, biobanking, remote monitoring of IC, clinical assessments and digital data collection. All data are collected in the INSPIRE-T database. Preventive strategies to limit errors like miscoding, missing values, are applied before data entry to ensure the validity and quality of the performed data analysis. Tools will be implemented for data exploration and data sharing between INSPIRE consortium researches and later on with external scientists.

### Objectives

The main objective of the INSPIRE-T cohort is the appropriate data collection of key variables and biospecimens for at least 1000 people at baseline and 800 people with at least four years of follow-up (i.e. four yearly post-baseline assessments) over the 10-year study. The key variables are clinical data on all six IC domains (locomotion, cognition, vision, hearing, vitality/nutrition, psychological status), and the collection of blood, urine, saliva and dental plaque samples. Secondary objectives include: i/the identification of (a set of) biomarkers of aging through the constitution of a comprehensive biobank; ii/the assessment of the feasibility and acceptability of the ICOPE Monitor app used to measure and monitor intrinsic capacity; iii/ the study of the evolution of IC domains over time and its association with health outcomes; and finally, iv/ the study of the correlation between digital biomarkers to biological/imaging biomarkers and IC domains (Figure 1, online consultation).

A mouse cohort in mirror of the human INSPIRE-T cohort is being built in order to cross the results of translational research found in humans on aging animal models, and vice versa (8). The main objective of the INSPIRE Animal cohort is to define the relationship between the molecular mechanisms of cell premature senescence and frailty/accelerated aging (8).

### Study population

We will recruit about 1000 subjects, men and women, aged 20 years-old or over (no upper limit for age), and affiliated to a social security scheme. People having a severe disease compromising life expectancy at 5 years (or at 1 year for subjects living in nursing homes) and people deprived of their liberty by administrative or judicial decision, or under guardianship, are excluded. Recruitment is stratified per 10-year age groups, oversampling older people in order to be able to investigate major clinical events (e.g., declines on IC, onset of age-related diseases).

Due to the heterogeneity of biological aging, we opted for no too stringent eligibility criteria. By diversifying our recruitment sources and monitoring key risk factors for accelerated aging (e.g., age, obesity, frailty, and activities of daily living), we will be able to recruit participants with different trajectories of aging.

Sample size calculation was not relevant as many objectives of the INSPIRE-T cohort are exploratory. We therefore considered an approach based more on the potential of the INSPIRE-T cohort in terms of the ability to obtain parameter estimates with sufficient precision with a recruitment of 1000 subjects that corresponds to the maximum number of subjects that can be recruited and monitored with the funding provided. In case of evident underpowered population (for a particular subgroup of subjects), a reasoned additional recruitment of subjects may be considered in a second phase. To limit the attrition rate, subjects will be monitored by both active (visits, telephone calls) and passive ways (monitoring of several functions using new technologies via mobile phones or other connected devices).

### Data collection

From all subjects enrolled, investigations include data collection at baseline and during follow-up visits (annual visits and additional visits planed in case of decline in IC). Upon written informed consent, the following set of information is obtained by using a standardized questionnaire:

- Demographic information: marital status, education, occupation and housing conditions, use of healthcare services;
- Physical examination comprising measurement of the following classical markers: medical history, medication, vaccination, current diseases, body mass index, waist and hip circumference, heart rate, blood pressure, self-reported visceral pain, skin elasticity (cutometer measurement), cutaneous itching/pruritus;
- Lifestyle information: physical activity, sedentarity time, smoking, alcohol consumption, solar exposure;
- Fried frailty phenotype (9);
- Functional status: Activities of Daily Living (ADL) (10) and Instrumental Activities of Daily Living (IADL)(11);
- Cognitive status: Mini Mental State Examination (MMSE) (12) and for people lower than 70 years, neuropsychological tests including free and total recall of the Free and Cued Selective Reminding Test (13), ten MMSE (12) orientation items, the Digit Symbol Substitution Test score from the Wechsler Adult Intelligence Scale—Revised (14), and the Category Naming Test (15) (2-minute category fluency in animals);
- Nutritional status: Mini Nutritional Assessment (MNA) (16), food frequency questionnaire (17);
- Oral status: Oral Health Assessment Tool (OHAT) (18);
- Depressive symptoms: Patient Health Questionnaire (PHQ-9) (19);
- Physical performance: Short Physical performance battery (20) and chair rise test (30 seconds)(21–22);
- Participant-reported outcome for cognition (CFI) (23) and mobility, fatigue, and social isolation (PROMIS) (24);
- Objective physical activity and sleep parameters (parameters are collected for one week using activPAL accelerometer);
- Vision: WHO simple eye chart, and the AMSLER Grid;
- IC domains (ICOPE Step 1) by using the ICOPE Monitor app. This app will be used throughout the study for the remote (at-distance) evaluation and monitoring (self-monitoring). All cut-offs operationalizing a deficit in IC comes from the WHO ICOPE program (4,7). At the first visit, the research team explains to the participants how to use the ICOPE Monitor app and monitor their IC domains over time. At each annual regular visit, the research team will confirm participants apply the correct evaluation procedures for assessing their IC. The 6 domains of IC evaluated by the ICOPE Monitor app are:
  - ○ Mobility measured by the time (in sec) spent to raise from a chair, at 5-repetition at a maximum speed. Declines will be considered when the time needed to complete the test is higher than 14 sec,
  - ○ Cognitive measured by the 3-word remember test of the MMSE (12) and the following questions: Do you have problems with memory or orientation (such as not knowing where one is or what day it is)? Did you notice a worsening of these disorders in the last 4 months or since the last evaluation? What is the full date today? (day, month, year, day of the week). For the 3-word remember test, three different sets of words will be used to avoid memory bias between two close assessments. Declines are present if the individual is unable to remember at least one word or if he/she provide a wrong response to the orientation question, ○ Psychological measured by the following two questions: Over the past two weeks, have you been bothered by: 1. Feeling down, depressed or hopeless? 2. Little interest or pleasure in doing things? One “YES” response determines a decline,
  - ○ Vitality/nutrition measured by the following two questions: Have you unintentionally lost more than 3 kg over the last 3 months? Have you experienced loss of appetite? One “YES” response determines a decline. One further question will be asked: what is your actual weight (in kilograms)?
  - ○ Sensorial-hearing measured by the Whisper test according to the following procedures: the evaluator must 1/stand about an arm’s length away behind and to one side of the person; 2/ ask the person or an assistant to close off the opposite ear by pressing on the tragus (the tragus is the projection in front of and partly covering the opening of the ear); 3/ Breathe out and then softly whisper a word with two syllables (a set of words will be selected by the Inspire research team), use a common word; 4/ Ask the person to repeat the word; and 5/ Move to the other side of the person and test the other ear, use a different word. Not repeating the correct words determines a decline. If the Whisper test can’t be realized, two questions are asked: Did you notice a worsening of these disorders in the last 4 months or since the last evaluation? Does your family complain an acute recent hearing loss?
  - ○ Sensorial-vision measured by the following questions: Do you have any problems with your eyes: difficulties in seeing far, reading, eye diseases or currently under medical treatment (e.g. diabetes, high blood pressure)? Declines are considered present when a person responds «yes» to this question and if she did not recently consult an ophthalmologist.

Other examinations are proposed to a limited number of participants (all age ranges and functional status) in a volunteer basis: Dual energy X-ray absorptiometry (DXA) for body composition assessment; Whole body and brain magnetic resonance (MRI); cardiorespiratory fitness (maximum oxygen consumption (V02 max) with blood sampling before and after the effort, and maximal aerobic power), and isokinetic muscle strength. These examinations are proposed annually for the DXA and, every two years for the other tests (MRI, V02 max, Isokinetic muscle strength). Participant-reported outcome for sarcopenia (SARQoL) (25) is completed for volunteers who perform cardiorespiratory fitness exploration.

### Digital assessments

Innovative digital assessments are also planned to be tested, such as home sensors (e.g., for measuring walking speed and its variability in daily environment), automated video analysis of mobility, and 3D facial images for the detection of digital markers of aging. A subgroup of 100 patients monitored by ambient sensors at home or sensors worn on the wrist over the long term will allow to remote and continuous monitoring of the trajectories of the IC domains (especially mobility, sleep parameters and nutrition) (CART/SmartHome research ancillary study, legal authorizations in process). This sub-study, developed in partnership with the CART research project team in United States (ORCATECH Team, Oregon Health and Science University, OR, USA; PI, Jeffrey Kaye; http://www.ohsu.edu) will allow us to detect subtle changes that are not clinically perceptible, well before the appearance of signs and symptoms and therefore determine innovative digital biomarkers and decision thresholds. These digital biomarkers will be correlated with clinical data but also biological and imaging biomarkers.

### Biobank

Biospecimens are collected during the INSPIRE-T cohort for the creation of a biobank.

Biospecimens, including blood, urine, saliva, dental biofilm, are collected from all subjects at baseline and then, annually (the genotyping sample will be collected only at the baseline visit). Nasopharyngeal swabs and cutaneous surface samples are collected from all subjects every two years. Feces, hair bulb, and skin biopsy, are collected optionally at baseline visit (see Table 2).

**Table 2:** Samplings proposed to the INSPIRE Human Translational Research Cohort participants for the creation of the biobank

**Biospecimens**	**At inclusion**	**Every Year**	**Every2years**	**Types of analyses**
Blood (60 ml each visit)	✓	✓		MultiOMICs analyses (proteomic, lipidomic, metabolomic, transcriptomic) SARS COV-2 infection
Nasopharyngeal sample	✓		✓	Carrying the respiratory syncytial virus, influenza virus and Clostridium difficile
Urine (20 ml at each visit)	✓	✓		MultiOMICs analyses
Saliva (10 ml at each visit)	✓	✓		Metabolomic Analyses, Proteomic Analyses
Dental plaque	✓	✓		Metabolomic Analyses Microbiome
Skin samples:				
‐swabbing, delamination	✓		✓	Proteomic and lipidomic analyses, Microbiome
‐skin biopsy (optional)	✓			Amplification and biobanking of human fibroblasts, generation of IPS cells
Hair bulb (optional)	✓			Proteomic/transcriptomic analysis Oxy-proteome
Feces (optional)	✓			Microbiome

Aliquots of biological material are stored at −80°C (dental biofilm, saliva, serum, plasma and urine) or at −196° C liquid nitrogen (PBMC) at the central lab (CRB TBR, CHU Toulouse/IFB PURP AN, Toulouse, France). Analysis will be either performed in Toulouse by the local biological teams involved in the INSPIRE project or by any third party not yet determined. The modality of Laboratory Data Transfer from the central lab to other parties will be defined at a later stage. Samples from the biobank may be moved to other US and European countries if required.

The INSPIRE-T biobank is supervised by the CRB TBR where all measures are taken to ensure a quality service based on appropriate resources and adequate safety procedures: observance of Good Laboratory Practice guidelines (the CRB TBR is certified AFNOR since 2015), fully-equipped premises, appropriate, approved and safe equipment (17 freezers −80°C Eppendorf Cryocube, 2 liquid nitrogen tanks with manual and documented filling, Vigitemp probes provide metrological tracking), qualified personnel, safety test and system implementation, sample traceability (all of biological collections are tracked in a specific software (TD Biobank), and CHU servers are daily backed up). All freezers are equipped with an alarm system. Equipments are monitored three times per day. Every failure is reported in the Kalilab software as non-compliance statements.

All the participants will be tested for SARS COV-2 infection via serological tests from blood collection when these latter will be available.

**Figure 2:**
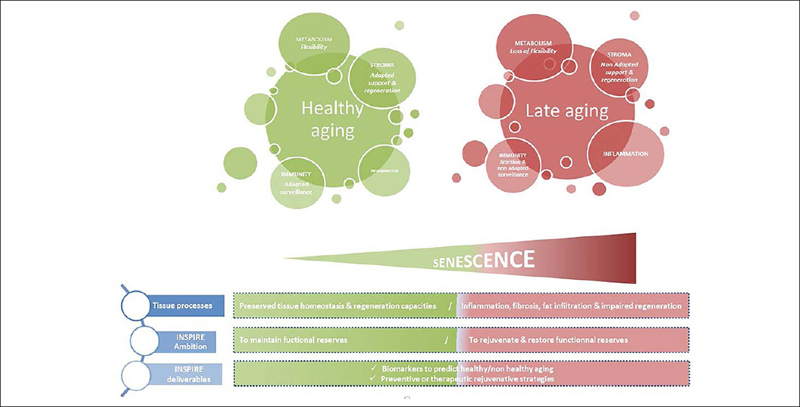
The INSPIRE approach: an integrative view of biological aging

All biological samples are processed within 110 min following a protocol elaborated for INSPIRE purpose and split into smaller aliquots at the INSPIRE-T biobank (Figures 2 & 3, online consultation).

#### Blood collection

For blood collection, all subjects are asked to donate blood (60 ml) by venipuncture after overnight fasting. The blood sample is processed to obtain whole blood, plasma, red blood cells, serum and peripheral blood mononuclear cells (PBMC).

For whole blood and serum, samples are immediately shipped after collection at room temperature to the CRB TBR for the preparation of whole-blood and serum aliquots for freezing at −80° in the INSPIRE-T biobank.

For PBMC, blood samples are immediately transported to the CRB TBR at room temperature and treated within 24 hours from time of collection. PBMC are collected after density gradient-based separation, counted and frozen at 8–14 millions/cells per vial. Frozen vials will be stored in liquid nitrogen.

For plasma and RBC (EDTA/Lithium Heparin/BDPIOO Blood), aliquots are immediately prepared after collection in the CRC and stored at −80°C until their shipment to the INSPIRE-T biobank.

When visits are organized by the mobile research team, some blood samples are not performed to limit quality procedures deviations (it concerns the Lithium Heparin tube, the 2 whole blood EDTA tubes and the BD P100 blood tube).

#### Urine collection

Participants are asked to collect at least 20 ml urine in a sterile screw-top container. The obtained volume is transferred into two vacutainer tubes of 10 ml each and directly shipped at room temperature to the CRB TBR where urine aliquots of 1 ml are prepared and stored at −80°C in the INSPIRE-T biobank.

#### Saliva

Participants are asked to collect 10 ml saliva in 50 ml Falcon tube (at least 30 min after a meal). The Falcon tube is immediately shipped at room temperature to the CRB TBR where saliva aliquots of 1 ml are prepared and stored at −80°C in the INSPIRE-T.

#### Dental biofilm collection

Biofilm sampling consists in recovering the biofilm from the external surfaces of the teeth (from natural teeth in priority, from prosthetic teeth if not possible), at the juxta-gingival level by curettage at 4 sites distributed over the dental arches: a sample from the upper anterior teeth, a sample from the upper posterior teeth, a sample from the lower anterior teeth, a sample from the lower posterior teeth. The product of each curettage is individualized in a sterile 1 ml cryotube. The four cryotubes are frozen at −80° in the CRC after the collection and regularly shipped to the CRB TBR.

#### Nasopharyngeal swabs

Nasopharyngeal swab will be addressed to the Institute of Biology of the CHU Toulouse within 4 hours from time of collection for their analysis (detection and identification of multiple respiratory viral and bacterial nucleic acids). Residuals samples will be stored at −80° to the CRB TBR in the INSPIRE-T biobank.

#### Skin swab and stripping

Swab samples are done on a skin exposed area (posterior face of the forearm) and a non-exposed area (lower back). Specimens are stored immediately at −80°C in the CRC. Frozen tubes are regularly shipped to the CRB and stored at −80°C in the INSPIRE-T biobank.

In addition, two 14 mm diameter D-squames are applied successively on the exact same area of the posterior face of the forearm: the first one is discarded and the second one is stored in a 2 ml tube. The same procedure is performed on the lower back. Both tubes are regularly shipped to the CRB and stored at −80°C in the INSPIRE-T biobank.

#### Feces collection (optional)

Feces are collected at the first visit and immediately stored at −80°C. If it is not possible, the participant can return to the research facility within one week with its frozen sample in a coproculture pot, placed in a cool box. The frozen feces samples are regularly shipped to the CRB TBR and stored at −80°C in the INSPIRE-T biobank.

#### Hair bulb collection (optional)

Twenty hairs are taken with the bulb and immediately stored after the collection in a sterile 2 ml cryotube in the CRC until their shipment to the INSPIRE-T biobank.

#### Skin biopsy (optional)

A 4 mm skin biopsy is obtained by using a punch. Skin samples are prepared according two different procedures: half of the samples are immediately rinsed, dried and stored at −80°C until its shipment to the CRB TBR; the other are immediately placed in a cryotube with PBS for cells cultures to organize a biobank of skin fibroblasts.

A biobank scientific committee will be set up, in the aim of determining the scientific directions and research priorities, of evaluating ongoing projects and their state of progress, and of resolving any methodological and ethical concerns raised by the studies. It shall i/examine the relevance, feasibility and conditions of implementation of the propositions concerning any analyses; ii/ ensure that national and international partnerships are made formal; iii/ control use of data, especially sample use, and iv/ ensure that participants rights are protected. The data disclosed will be made anonymous (coded, traceable data).

### Statistical methods

Since the primary outcome measure of the INSPIRE-T cohort is related to reaching prespecified numbers for recruitment and retention, we will use numbers and percentages. Hypothesis-testing statistics will be employed for some of the secondary outcome measures and the new hypothesis arising through the 10 year duration of the INSPIRE program. Specific statistical analysis plan (SAP) will be written to answer each research question. Big data methods of analysis will be considered when examining the large and diversified amount of data that will be gathered from clinical and para-clinical evaluations, biospecimens, and digital assessments.

Significance will be set at p ≤ 0.05. Analyses will be performed using Stata (v14, StataCorp), SAS (v9.4, Cary, NC, USA), and R (v3.5.2). Statistical analyses will be done by researchers of the INSPIRE program and professional statisticians. Analyses by gender will be conducted.

### Ethical and regulatory considerations

The INSPIRE-T cohort is carried out in accordance with the declaration of Helsinki, which is the accepted basis for clinical study ethics, and must be fully followed and respected by all engaged in research on human beings. The INSPIRE-T cohort protocol has been approved by the French Ethical Committee located in Rennes (CPP Ouest V) in October 2019. This research has been registered on the site http://clinicaltrials.gov (ID NCT04224038).

## Current progress of the INSPIRE -T cohort

### Recruitment status

The first participant was recruited on October 16 2019. Our objective is to recruit at least, 1000 people at baseline (500 during the first year and 500 during the second year of the project) from 20 onwards, including robust, prefrail and frail older adults, as well as disabled people, to be able to better understand the biology of aging across age-ranges and functional status. All the recruiting work is currently carried out by the Toulouse Gerontopole research team on a single site; a mobile clinical research team is also currently active to recruit frailer population (e.g. people unable to come to research facilities) in Toulouse and surrounding areas. Current inclusion rates are 4 participants per day. This rate will allow us to reach our objective of 1000 inclusions in 2 years. Two hundred and forty participants have been included by March 13 2020 (137 women / 103 men; mean age: 74.6 years), and 400 new inclusions are planed until September 2020. Among the 240 enrolled participants, 168 are robust, 60 prefrail and 6 frail with Fried criteria (9). All participants gave their consent for the complete biobanking, 112 participants have accepted the skin biopsy, 231 hair bulb collection and 216 feces collection. All subjects have accepted the DXA, and 211 V02 max and muscle strength assessment. The sub-study on MRI is planned to start on September 2020. However, recruitment has been temporarily suspended during the COVID-19 pandemic.

### Recruitment strategies

Our first plans were to recruit a representative sample of users of primary care services, by inviting people to participate using patients’ list of several family physicians in different areas (with different deprivation levels) of Toulouse (all patients aged 20 years or over being invited to be screened for participation). However, this recruitment approach proved to be unfeasible, mainly because many physicians have been very busy taking care of several viral pathologies during winter 2019–2020 (including COVID-19 from February 2020) (26, 27). Consequently, we decided to diversify the sources of recruitment.

Current recruitment relies mainly on the following strategies: flyers, community outreach strategies, media coverage, newspaper advertising, posters, online promotion, mass mailing, presentations at public events, conferences, study website, dissemination through institution newsletters, identification of participants from previous studies or existing registries, onsite recruitment /medical records review (by investigators/clinical research assistants), dissemination through health care providers : coordination with primary care, memory centers, hospital outpatient clinics, medical centers, physicians (site investigators, primary care physicians), specialists, hospital inpatient lists, private clinics, and finally, dissemination through residential homes, and nursing homes. The recruitment channels of the participant included (and planed over the next 6 months) is detailed in Table 3. Applied strategies are constantly followed and adapted if necessary throughout the recruitment study period (weekly meeting with investigators and study staff).

**Table 3:** Recruitment strategies implemented in the INSPIRE Human Translational Cohort

**Recruiting Source**	**Recruited participants (n, %)**	**Planned visits (n,%)**
Medias	85 (35.4)	199 (49.2)
Online Promotion	32(13.3)	33 (13.7)
Hospital outpatients clinics	45 (18.7)	42 (10.4)
Public conferences	25 (10.4)	16 (6.7)
General Practitioners	10 (4.2)	9 (3.7)
Personal contact	33 (13.7)	82 (20.3)
Residential homes, nursing homes	7 (2.9)	0
Others	3(1.2)	23 (5.7)
Total	240	404

A mobile research team was implemented in January 2020 to recruit frailer population by collaborating with residential homes, long-term care facilities and post-acute and rehabilitation facilities. Next collaborations are considered with the CRCT in Oncopole-Toulouse (an institution dedicated to cancer research and care) and a private clinic focused on the management of obese people.

Retention strategies are implemented in parallel. It consists of participant-centered values and strategies including (but not limited to) identify proxy contacts, minimize waiting time during study visits, facilitate transportation from and to research facilities, adapt comfortable waiting room facilities, build relationships with study participants; remind nonresponsive participants (contact via phone or email, make phone calls during optimal hours; offering regular feed-back during the follow-up (mailing study updates); offering regular gadgets during the follow up and postcards.

### Perspectives

The INSPIRE-T cohort will gather clinical, biological (including imaging), and digital data for subjects of several chronological ages and functional capacity status regularly followed over up to 10 years. One of the most innovative aspects of the INSPIRE-T cohort is that, through a close monitoring of participants with the ICOPE Monitor app, we will obtain clinical and biological data at the moment declines in IC come up. The cohort will provide us the needed resources to improve our understanding of the biological mechanisms of aging and the natural history of loss of IC leading to dependency during aging. By following and monitoring the IC of participants over time, this study will provide information about a new, function-centered healthcare pathway, which would agree with WHO recommendations for an integrated care for older people. At the medium term, this data may inform the development of a pragmatically interventional study testing the effects of this new healthcare pathway on clinical outcomes in older people; this healthcare pathway may be integrated in daily practice in healthcare systems, becoming thus the usual care. Innovative digital solutions (including sensors) proposed in the INSPIRE-T cohort are a promising way to remotely collect and analyze real-life and continuous health related data and thus longitudinal trajectories over time. It makes it possible to detect subtle variation in the IC before a clinical event.

The INSPIRE-T cohort will also perform relevant and extensive bio banking to allow basic and translational research in humans in the field of Geroscience and Healthy Aging. The INSPIRE-T biobank might lead to improving our understanding about molecular and physiological mechanisms involved in healthy aging, interacting with changes linked to specific chronic diseases. This may contribute to establish a set of biomarkers, that could be pharmacologically or non-pharmacologically targetable, and that would characterize biological aging and, then, permit to identify an accelerated aging phenotype. In their recent paper, Ahadi S et al (28) have defined different types of aging patterns in different individuals, termed “ageotypes”, on the basis of the types of molecular pathways that changed over time in a given individual. According to the authors, “ageotypes” may provide a molecular assessment of personal aging, reflective of personal lifestyle and medical history that may ultimately be useful in monitoring and intervening in the aging process”. One of the main objectives of the INSPIRE-T cohort is to identify biological markers that could detect the inter-individual variability of biological processes before it becomes clinically perceptible (29). The identification of biomarkers of aging may help us to identify individuals who are with a high risk of developing age-associated diseases, decline in IC or disability, and to propose personalized strategies, including innovative therapeutics, to prevent or restore impaired functions. Our clinical and biological data will give the opportunity to explore the interaction between changes with aging on inflammation, metabolism, gerosciences in general, and neurodegenerative process leading to Alzheimer’s disease (30, 31) or physical frailty (32), two major causes of loss of functions. The INSPIRE-T cohort will benefit from the availability of plasma neurodegenerative biomarkers (plasma amyloid beta 42/40, neurofilaments, plasma phospho tau) (31). The development of biological markers of frailty is also required to improve the treatment of frail individuals. The etiology of frailty is complex. Proposed biomarkers of frailty include markers of inflammation. As recently proposed by the ICSFR Task Force perspective on biomarkers for sarcopenia and frailty, «machine learning and information technology innovation could thus be used to develop risk scores that could be used in clinical and research settings. Other technologies, such as induced pluripotent stem cells (iPSCs) or skin fibroblasts, could be used to study markers of senescence and could also enable a move towards personalized medicine» (32). Interventions to promote healthy aging will be more effective in people with a risk of decline (29). Hallmarks of aging are under scrutiny in particular DNA alteration, epigenetics, unusual protein production, senescent cells secreting pro-inflammatory factors and others. New therapies aim to target senescent cells or their secretory proteins (the senolytic molecules) and therefore promote healthy ageing are presently under development (33–36).

Finally, the INSPIRE-T cohort gives us the opportunity to federate clinical and biological research teams in Toulouse and Occitania Region to build a research platform of gerosciences discovery to explore mechanisms of aging, and to implement comprehensive translational projects towards the goal of preventing the consequences of aging for a healthy, and long-lived society (37). The animal cohort, generated to “mirror” the human translational cohort, will facilitate the translation of results from basic research to humans and to the clinics. The identification of markers of aging will take advantage of three complimentary approaches to look for the best markers of aging: without a priori approaches (transcriptomics, proteomics, metabolomics); semi a priori approach (metabolism, inflammation, cell cycle, mitochondrial network …); and targeted approach (pre-identified targets such as (but not limited to) Growth Differentiating Factor 15 (GDF-15), apelin, senescent cells, amyloid protein in plasma) (38). From a biological viewpoint, the function-centred approach recommended by WHO represents a challenge due to the multidimensionality that characterizes IC trajectories during aging. We will develop an integrative view of biological aging (Figure 2). Three classes of parameters transversal to the whole organism, present in all organs, strongly interrelated and crucial in tissue homeostasis have been selected: i) inflammation and immunity that represents both a warning signals and the house keeping guard of tissue integrity, ii) mesenchymal stem/stroma cells (MSC) allowing support for all function and their adaptation and iii) metabolism that controls any cell decision and the fate of most of them. For all these transversal components, senescence mechanisms will be carefully investigated.

Due to the Covid19, teleconsultation has been added for the pre-inclusion and some of the assessment, we will be able also to assess the “stay at home order” on the INSPIRE-T cohort subjects (39).

In conclusion, the INSPIRE-T cohort, nested in the INSPIRE Platform, will contribute to healthy aging and dependency prevention. The INSPIRE-T cohort will foster discoveries of human markers (i.e., biological, clinical, digital) of healthy aging capable of predicting functioning and resilience.

## Electronic supplementary material

Supplementary material, approximately 504 KB.
