# Supplementary material for: The INSPIRE Bio-Resource Research Platform for Healthy Aging and Geroscience: Focus on the Human Translational Research Cohort (The INSPIRE-T Cohort)
Source: J Frailty Aging. 2020 Jul 10;10(2):110–20. doi: 10.14283/jfa.2020.38 (PMC7352084; doi:10.14283/jfa.2020.38)
Supplement: Supplementary file 1 — Supplementary material, approximately 504 KB. [file 42415_2020_38_MOESM1_ESM.docx]

#### Authors


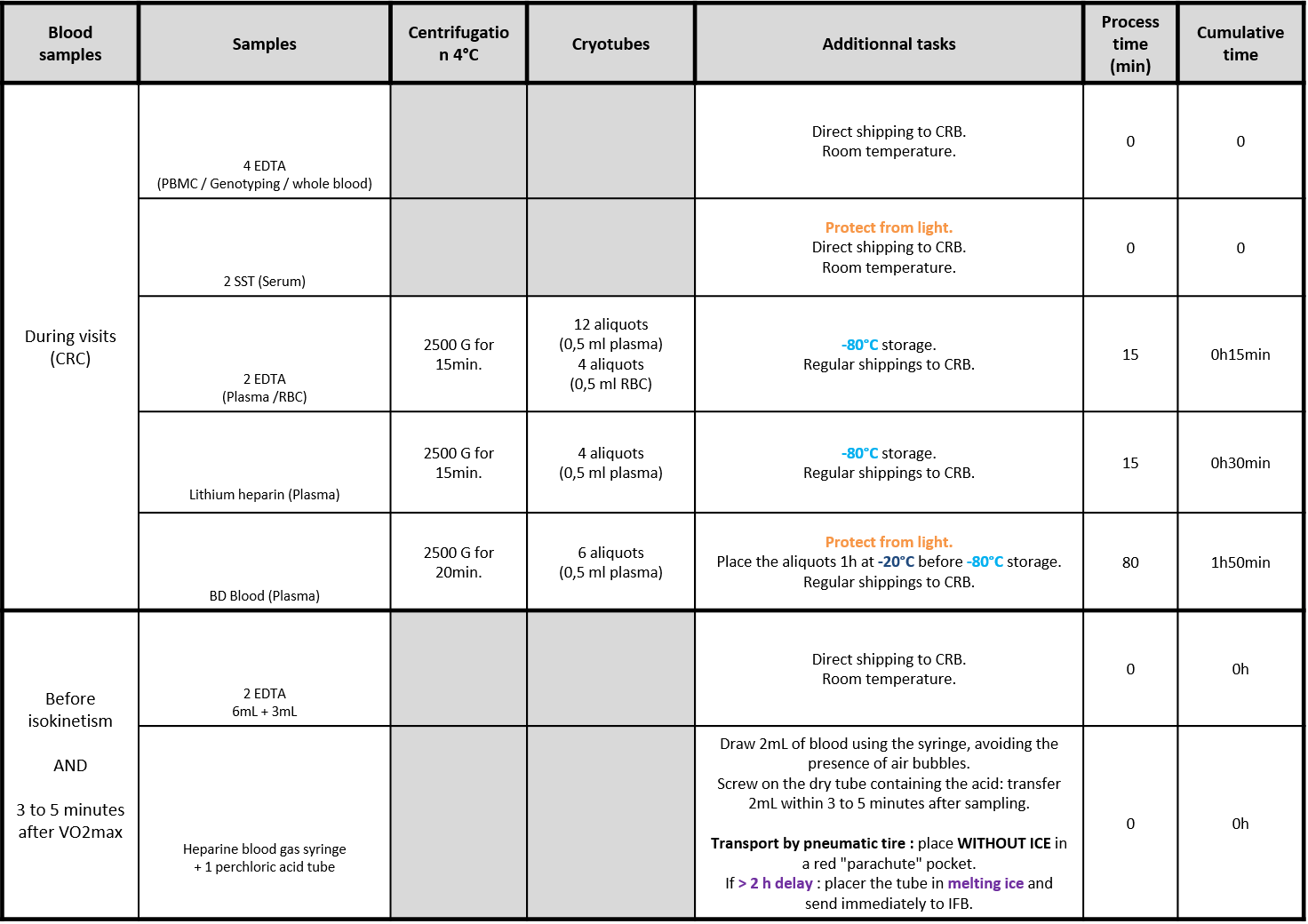
ONLINE CONSULTATION - Figure 2. Laboratory procedures for preparation of biological material (blood samples)


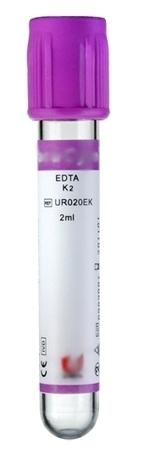

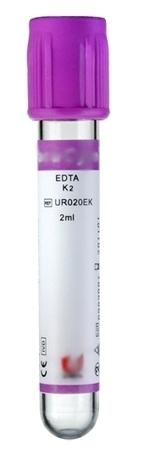

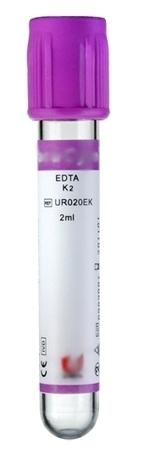

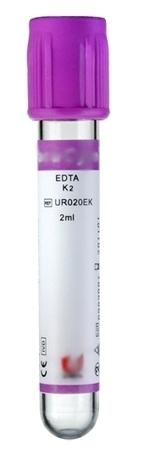


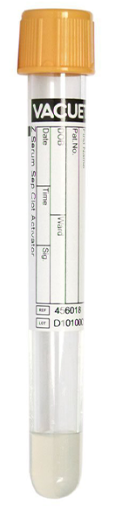

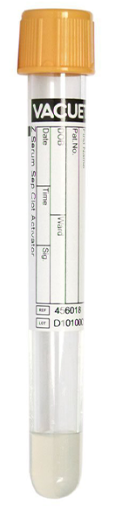


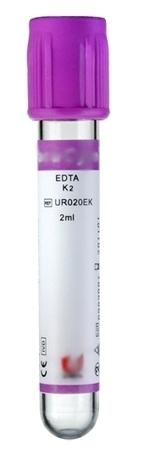

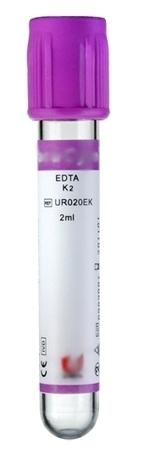


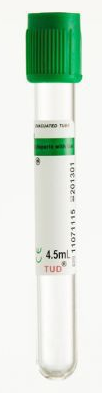


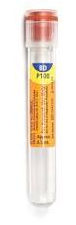


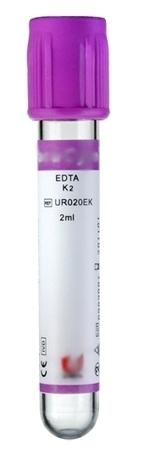

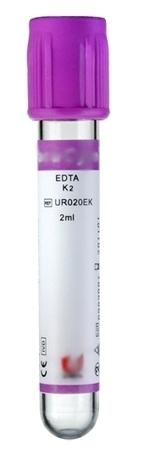


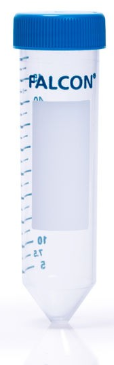

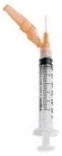


ONLINE CONSULTATION - Figure 3. Laboratory procedures for preparation of biological material (skin, dental plaque, saliva, urine, nasopharyngeal)


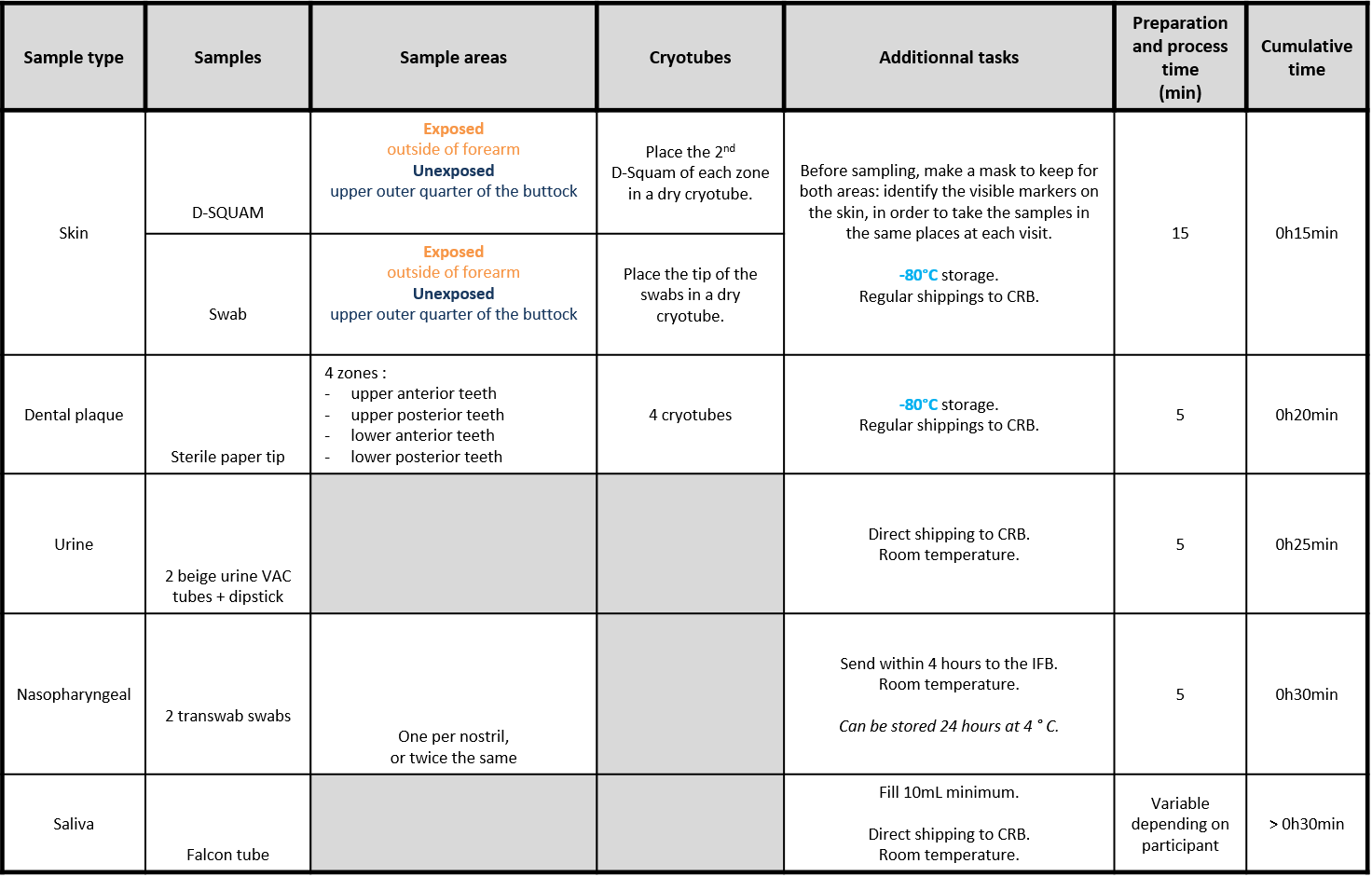

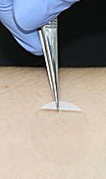

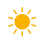

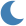

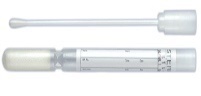

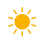

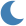

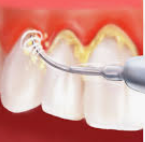

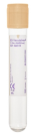

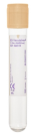

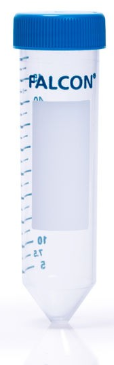

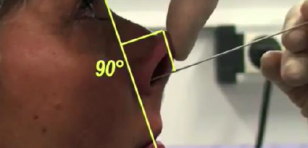

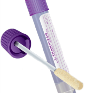

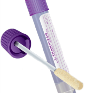


ONLINE CONSULTATION – Figure 1. Organizational chart describing the various study components of the INSPIRE-T cohort

**Scientific Committee of**

**the INSPIRE Program**

**Financial Management Team**

**Regulatory Management Team**

**Legal Management Team**

In charge of :

1/ Conduction of the study :

Recruitment and retention of subjects

Clinical, biological and digital collection

Remote monitoring of IC

2/Implementation of the standardized operational procedures

3/ Preventive strategies to ensure data quality

**Technical and operational forms**

Biological Resource Center « CRB TBR » (supervision of the INSPIRE-T biobank)

Clinical Research Imaging (supervision of imaging data)

University sports Clinic (supervision of cardiorespiratory fitness exploration)

Clinical Research Center (CRC)/ Research mobile team

Unity of support in research methodology

**Project Management Team of the INSPIRE T Cohort**
